# Antitumor Activity of Royal Jelly and Its Cellular Mechanisms against Ehrlich Solid Tumor in Mice

**DOI:** 10.1155/2022/7233997

**Published:** 2022-04-11

**Authors:** Aishah E. Albalawi, Norah A. Althobaiti, Salma S. Alrdahe, Reem Hasaballah Alhasani, Fatima S. Alaryani, Mona N. BinMowyna

**Affiliations:** ^1^Department of Biology, Faculty of Science, University of Tabuk, Tabuk, Saudi Arabia; ^2^Biology Department, College of Science and Humanities-Al Quwaiiyah, Shaqra University, Al Quwaiiyah 19257, Saudi Arabia; ^3^Department of Biology, Faculty of Applied Science, Umm Al-Qura University, Makkah, Saudi Arabia; ^4^Biology Department, Faculty of Sciences, University of Jeddah, Jeddah, Saudi Arabia; ^5^College of Applied Medical Sciences, Shaqra University, Shaqra, Saudi Arabia

## Abstract

**Objective:**

The present study was aimed at evaluating the antitumor effects of royal jelly (RJ) obtained from *Apis mellifera* compared with cyclophosphamide against the Ehrlich solid tumors (EST) in mice.

**Methods:**

Tumor growth inhibition, body weight, the serum level of alpha-fetoprotein (AFP) and carcinoembryonic antigen tumor (CAE), liver and kidney enzymes, tumor lipid peroxidation (LPO), nitric oxide (NO), antioxidant enzymes (glutathione peroxidase (GPx), catalase enzyme (CAT), and superoxide dismutase enzyme activity (SOD)), tumor necrosis factor alpha level (TNF-*α*), and the apoptosis-regulatory genes expression were assessed in EST mice treated with RJ (200 and 400 mg/kg orally once a day for 2 weeks).

**Results:**

The results showed that treatment of EST-suffering mice with RJ at the doses of 200 and 400 mg/kg causes significant reduction in tumor volume and inhibition rate, body weight, tumor markers (AFP and CEA), serum level of liver and kidney, LPO and NO, TNF-*α* level, as well as the expression level of Bcl-2 in comparison with the EST mice receiving the normal saline; whereas RJ at the doses of 200 and 400 mg/kg/day significantly increased (*p* < 0.05) the level of antioxidant enzymes of GPx, CAT, and SOD and the expression level of caspase-3 and Bax genes.

**Conclusion:**

The findings revealed that oral administration of royal jelly especially at the doses of 200 and 400 mg/kg exhibited promising antitumor effects against EST in mice through induction of apoptosis as well as its antioxidant and anti-inflammatory effects, which suggest it as a novel anticancer agent against tumor; however, additional surveys especially in clinical setting are necessary to approve these findings.

## 1. Introduction

Cancer as one of the main concerns in public health is described by unregulated cell growth, invasion and spread of cells from the original site, or the unusual site in other parts of the body [1]. Cancer also is a main cause for death involving more than 7.5 million people around the world each year [2]. Previous reports have demonstrated the six main issues in the occurrence of cancer including the ability to produce messages of autonomic growth, escape growth inhibition messages, avoid apoptotic cell death, unlimited replication, angiogenesis, invasion, and metastasis [3, 4].

At present, a number of approaches are applied for the diagnosis and treatment of several types of cancer, the most significant of which are chemotherapy with chemical agents, radiotherapy, surgery, etc. [5]. By chemotherapy, there are several synthetic agents for cancer therapy including antimetabolites drugs (e.g., methotrexate), passive compounds of DNA (e.g., doxorubicin and cisplatin), antitubulin drugs (e.g., taxis), etc. [6, 7]. Recent reports have revealed some limitations and adverse side effects in the use of the synthetic agents, such as hair loss, nausea, vomiting, gastrointestinal and kidney disorders, bone marrow suppression, and fatigue, as well as the resistance of cancer cells to common therapies; these reasons encourage the researchers to search new agents especially in natural products with greater effectiveness and less toxicity [6, 7]

Natural products are well known as a valuable resource of appropriate new agents with great chemical diversity observed in millions species of herbs, animal, marine, and microbes [8]. In the recent decades, rising interest has observed on the part of consumers and the food industry into useful food materials and the habits in which it can help keep human health; whereas the key role of diet broadly has been reported for treatment and prevention of a large number of diseases such as cancers [9]. Between foods that have health-promoting properties are products deriving from the beehive, such as royal jelly, honey, and propolis [10].

Royal jelly (RJ) is one of the most noteworthy nutritious substance which is secreted from the hypopharyngeal and mandibular salivary glands of young nurse honey bees [11]. RJ due to having different bioactive compounds, such as polyphenols, protein, lipids, carbohydrates, and mineral salt, has various biological and pharmacological properties such as antioxidant, neurotrophic, anticancer, anti-inflammatory, antidiabetic, antilipidemic, and antimicrobial [11, 12]. Clinical trials, in vitro and in vivo studies, demonstrated that RJ displayed its anticancer effects through induction of apoptosis, thereby increasing the activities of antioxidant factors, inhibiting elevated serum markers and histological alterations, and regulating the inflammatory factors, etc [13]; therefore, the present study was aimed at evaluating the antitumor effects of royal jelly obtained from *Apis mellifera* compared with cyclophosphamide (CP), as an alkylating agent which widely used for the treatment of neoplastic cancers, against the Ehrlich solid tumors (EST) in mice.

## 2. Materials and Methods

### 2.1. Royal Jelly

To ensure the relative purity and authenticity of botanical sources, the royal jelly materials were obtained from May 2021 from Langstroth hives containing colonies of the *A. mellifera* grown at the Shaqra University, Saudi Arabia. RJ samples were dissolved with normal saline and filtered under a vacuum by means of filter paper (Whatman membrane, England) to reach the doses of 200 and 400 mg/kg. The selection of these concentrations was based on the previous study that showed promising biological effects with minimum toxicity [14–16].

### 2.2. Secondary Metabolites Contents

#### 2.2.1. Total Phenol Content

The total phenol content in RJ sample was assessed based on the technique explained previously. To do this, 50 *μ*L of RJ solution (50 mg/mL) was add to 250 *μ*L of Folin-Ciocalteu's reagent (0.2 N) for 6 minutes; then, 0.2 mL of sodium carbonate (7.5%) was put into the tested tubes. After 120 min incubation in room temperature, the absorbance of suspension was determined at 760 nm. Distilled water was also used as the blank solution [17].

Total phenol content was exhibited as mg gallic acid equivalents (GAE)/g. The determination of flavonoid content was performed according to the method explained by El-Guendouz et al. In this way, 200 *μ*L of RJ solution was poured into the tubes containing 200 *μ*L aluminum chloride (20%). The mixture was incubated at room temperature for 60 min, and the absorbance of it was determined at 420 nm [18].

#### 2.2.2. Total Flavonoid Contents

The total flavonoid contents were reported as milligram of quercetin equivalents per gram of RJ (mg QE/g RJ) by means of a standard curve. Here, we used the Bradford technique which also called Bio-Rad assay to determine the protein content. In brief, 200 mg RJ sample was added in a tube containing 10 mL methanol/water (50/50; *v*/*v*); the suspension was sonicated for 1 h. in the next step, the pH of suspension was adjusted to 2.5 with phosphoric acid and was then diluted 10 times. Next, 5 mL of Bio-Rad reagent was mixed with 250 *μ*L of RJ solutions. Lastly, the absorbance of mixture was determined at 595 nm. The total protein content was reported as percentage (%) by means of the bovine serum albumin standard curve [19].

### 2.3. The Ehrlich Ascites Tumor (EAT) Cell Line

Ehrlich ascites tumor (EAT) cell line were prepared from the American Type Tissue Culture Collection (Manassas, USA). EAT cells were then adjusted into 2 × 10^6^ cells/mL in sterile saline solution by means of a Neubauer hemocytometer.

### 2.4. Animals

A total of 56 female Swiss albino mice with weight of 20-25 g and 6–8 weeks' old were applied to induce the animal model of EST. Animals were kept in the animal house at 22–24°C with condition of 12 h light/12 h dark cycle. Animals also were provided with food for rodents and water ad libitum. This in vivo study was accomplished in consistent with the guidelines of the Guide for Care and Use of Laboratory Animals of the National Institutes of Health. However, the study was permitted by the ethical committee of Shaqra University, Saudi Arabia (214-2020).

### 2.5. Study Design

EST in mice were established by intramuscularly injection of 200 *μ*L of cell suspension in the right thigh of mice. On 7^th^ day after induction of the EAT, the mice were randomly divided into 7 groups (8 mice/group) including

- Non-EST and nontreated mice (C1)
- EST mice receiving the normal saline (C2)
- EST mice treated with CP (50 mg/kg) intraperitoneally once a day for 3 days (C3)
- Non-EST mice treated with RJ 200 mg/kg orally once a day for 2 weeks (C4)
- Non-EST mice treated with RJ 400 mg/kg orally once a day for 2 weeks (C5)
- EST mice treated with RJ 200 mg/kg orally once a day for 2 weeks (Ex1)
- EST mice treated with RJ 400 mg/kg orally once a day for 2 weeks (Ex2)

### 2.6. Blood and Tumor Sampling

On the 15^th^ after treatment, animals were anesthetized with ketamine (100 mg/kg) xylazine (10 mg/kg) by opening their abdomen cavities, and blood specimens were obtained from the animal's heart. Blood specimens were then centrifuged at 6000 rpm for 10 min and the acquired sera were separated and stored at −80°C until examining. Tumors were also aseptically collected and weighed, and their dimensions were recorded. After that, they were equally divided into two parts: one part was kept in −80°C for molecular examinations, and the other part was stored at −20°C for other examinations.

### 2.7. Tumor Growth Inhibition.

In this survey, by calculating the tumor volume (TV) and tumor growth inhibition rate, we assessed the in vivo antitumor activity of RJ. TV was determined by the Vernier caliper after the 7th day of the treatment through the equation TV (mm^3^) =4*π*(A/2)^2^ × (B/2); whereas A and B are the minor and major tumor axes. Tumor growth inhibition rate (TGIR) also was determined according to the technique elucidated elsewhere with the below formula [20]:

(1) $$\begin{array}{c} TGIR\;\left(\%\right)=\frac{\left(\text{The }mean\;tumor\;weight\;\text{of }control\;group–\text{the }mean\;tumor\;weight\;\text{of }treated\;group\right)}{\left(\text{The }mean\;tumor\;weight\;\text{of }control\;group\right)}\times 100. \end{array}$$

### 2.8. Body Weight (BW) Changes

Mice in all studied groups were weighed on day 7 and day 21. The percentage weight gain was determined using the following equation [18]:

(2) $$\begin{array}{c} \%\text{weight gain}=\left[\frac{\text{Mice weight on }19\text{th day}}{\text{Mice weight on day }0}\right]-1\times 100. \end{array}$$

### 2.9. Assessing the Tumor Markers

The alpha-fetoprotein (AFP) level in sera was evaluated by means of an automated quantitative enzyme-linked fluorescent assay (ELFA) using mini-VIDAS® AFP (bioMérieux, Marcyl'Etoile, France) according to the guidelines of the manufacturer. On the other hand, carcinoembryonic antigen tumor (CAE) level in mice sera was assessed by using quantitative sandwich immunoassay, MyBio-Source Mouse Carcinoembryonic Antigen Elisa Kit (MyBio-Source, San Diego, USA).

### 2.10. Evaluation of Serum Levels of Liver Enzymes

Liver function following treatment with RJ was assessed by measuring the serum level of alanine aminotransferase (ALT) and aspartate aminotransferase (AST) using the commercial diagnostic kits (Roche, Germany) [21].

### 2.11. Evaluation of Serum Levels of Kidney Enzymes

Kidney function following treatment with RJ was also determined by measuring the serum level of creatinine (Cr) and blood urea nitrogen (BUN) using the commercial diagnostic kits (Roche, Germany) [21].

### 2.12. Evaluation of the Oxidative Stress Markers

The level of oxidative stress (lipid peroxidation (LPO) and nitric oxide (NO)) in tumor homogenates was evaluated by a biodiagnostic analyze kits based on the malondialdehyde (MDA) creation through the thiobarbituric acid (TBA) procedure defined by of Ohkawa et al. [22]. No production was also evaluated in the tumor homogenates based on the method explained by Green et al. [23].

### 2.13. Evaluation of the Antioxidant Enzymes

The antioxidant activities were assessed through the evaluation of some enzymes such as glutathione reductase (GR), glutathione peroxidase (GPx), catalase enzyme (CAT), and superoxide dismutase enzyme activity (SOD) by means of the commercial kits and according to the technique defined by Weydert and Cullen [24], Luck [25], and Sun et al., [26], respectively.

### 2.14. Measuring the Tumor Necrosis Factor Alpha Level (TNF-*α*)

Here, we evaluated the level of TNF-*α* in tumor homogenates by means of mice TNF*α* ELISA kit (ab100747; Abcam) based on the guidelines of the manufacturer.

### 2.15. Assessing the Expression Level of Apoptosis-Regulatory Genes

The expression level of some apoptosis-regulatory genes such as caspase-3, Bcl2, and Bax was evaluated by quantitative real-time PCR. Briefly, a total RNA of tumor tissue was extracted by an RNeasy tissue kit (Qiagen, Germany) in line with the protocols of manufacturer. Then, cDNA synthesis was produced using random primers through the complementary DNA (cDNA) synthesis based on the manufacture's recommendations. In the next step, cDNA was utilized for conventional PCR reaction analysis or real-time PCR through SYBR green. The thermal profile of reaction was 95°C for 8 min, 40 cycles of 95°C for 10 s, and 56°C for 30 s, respectively. Finally, the *Δ*Ct was calculated by means of iQTM5 optical system software (Bio-Rad, Hercules, CA). *β*-Actin was applied as a housekeeping gene and normalization control.  Table 1 displayed oligonucleotide primers which were used for real-time PCR [27].

### 2.16. Statistical Analysis

All obtained results were presented as the means ± standard deviation. SPSS statistical software version, 22.0 (SPSS Inc., Chicago, IL, USA) was applied for data analysis. One-way ANOVA with Turkey's potshot test was used to assess the differences between the experimental groups.

## 3. Results and Discussion

Cancer as a main concern of public problem is one of the most serious causes of death among humans [2]. In recent years, studies have demonstrated some limitations and adverse side effects of existing drugs in the treatment of cancer; these reasons promote the researchers to search for substitute anticancer agents with relevant efficacy and fewer toxicity [5].

RJ, a viscous secretion of A. mellifera worker bees, is considered an important functional natural product with a wide range of commercial, cosmetic and medical applications [10]. Moreover, in modern medicine, RJ has been demonstrated various pharmacological properties such as antioxidant, anti-inflammatory, immunomodulatory, nephroprotective, wound-healing, and antimicrobial effects [11–14]. The present study was aimed at evaluating the antitumor effects of royal jelly obtained from *A. mellifera* compared with cyclophosphamide (CP), as an alkylating agent which is widely used for the treatment of neoplastic cancers, against the Ehrlich solid tumors (EST) in mice.

The findings of the secondary metabolites analysis of RJ displayed that total phenolic and flavonoid content was 96.3 ± 0.31 (mg GEA/g DW) and 2.85 ± 0.026 (mg QE/g DW), respectively; the results also showed that the total protein content of RJ sample was 11.3% (Table 2).

**Figure 1:**
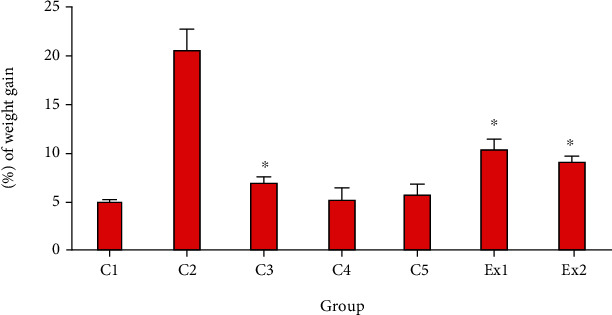
The mean of % of weight gains in EST-bearing mice treated with royal jelly (RJ). Non-EST and nontreated mice (C1); EST mice receiving the normal saline (C2); EST mice treated with CP (50 mg/kg) intraperitoneally once a day for 3 days (C3); non-EST mice treated with RJ 200 mg/kg orally once a day for 2 weeks (C4); non-EST mice treated with RJ 400 mg/kg orally once a day for 2 weeks (C5); EST mice treated with RJ 200 mg/kg orally once a day for 2 weeks (Ex1); EST mice treated with RJ 200 mg/kg orally once a day for 2 weeks (Ex2). Data are expressed as the mean ± SD (*n* = 8). ^∗^*p* < 0.001 significant difference compared with C2 group.

As shown in Table 3, treatment of EST-suffering mice with RJ meaningfully reduced the tumor volume in a dose-dependent response; RJ at the doses of 200 and 400 mg/kg decreased the tumor weight by 1.54 ± 0.04 g and 0.86 ± 0.022 g, respectively. The findings also revealed tumor inhibition rate was 50.6 and 72.4% after treatment of EST-suffering mice by RJ at the dose of 200 and 400 mg/kg, respectively. By the body weight evaluation, as shown in Figure 1, EST-suffering mice treated with RJ (200 and 400 mg/kg) displayed a significant (*p* < 0.05) decrease in BW when compared with that of untreated EST mice in the C2 control.

In regard to anticancer effects of royal jelly, Nakaya et al. have shown that RJ has anticancer effects by suppression of the estradiol-induced cell proliferation of MCF-7 breast cancer cells [28]. Mohammadi Abandansari et al. have reported that RJ especially at the concentrations of 50 and 100 mg/mL significantly showed the cytotoxic activity against the prostate cancer cell line [29]. Recently, in a randomized double-blind clinical trial, Miyata et al. have revealed that oral administration of capsules having 900 mg royal jelly considerably reduces the tumor size and some adverse side effects such as fatigue and anorexia in patients with renal carcinoma [30]. In the other study conducted by Zhang et al., the results exhibited that RJ at the doses of 0.5 and 1.5 g/kg meaningfully decreased the tumor weight in the 4 T1 (breast tumor)-suffering mice [31]. In addition, previous clinical trials, in vitro and in vivo studies, demonstrated that RJ displayed its anticancer effects through induction of apoptosis, thereby increasing the activities of antioxidant factors, inhibition of elevated serum markers and histological alterations and regulation the inflammatory factors, etc [13].

It has been previously proven that RJ contains several bioactive compounds such as peptides, proteins, fatty acids (e.g., 10-hydroxydecanoic acid), polyphenols, and flavonoids (e.g., pinocembrin, quercetin, and galangin) [32]; while in other studies, the anticancer effects and possible mechanisms of these compounds have been proven. For example, Bhosale et al. have demonstrated that the polyphenols compounds display their anticancer activity through several mode of actions such as elimination of cell via signaling pathways alteration, suppression of cell cycle actions, and apoptosis stimulation as well as their antimetastasis, antiangiogenic, etc [33]. Kopustinskiene et al. also have shown that flavonoids exhibit their anticancer effects through a number of mechanisms such as autophagy, controlling of reactive oxygen species- (ROS-) scavenging enzyme activities, suppressing the cell cycle, and promoting the apoptosis and inhibition of proliferation of cancer cells [34]. Recently, Albalawi et al. have reported that queen bee acid (10-hydroxy-2-decenoic acid, 10-HDA), a main fatty acid in royal jelly, at the doses of 2.5 and 5 mg/kg mainly in combination with cyclophosphamide, displayed favorable antitumor activity against EST in mice through induction of apoptosis and increasing the antioxidant activities and be able to be suggested as novel substitute anticancer agent [35].

**Figure 2:**
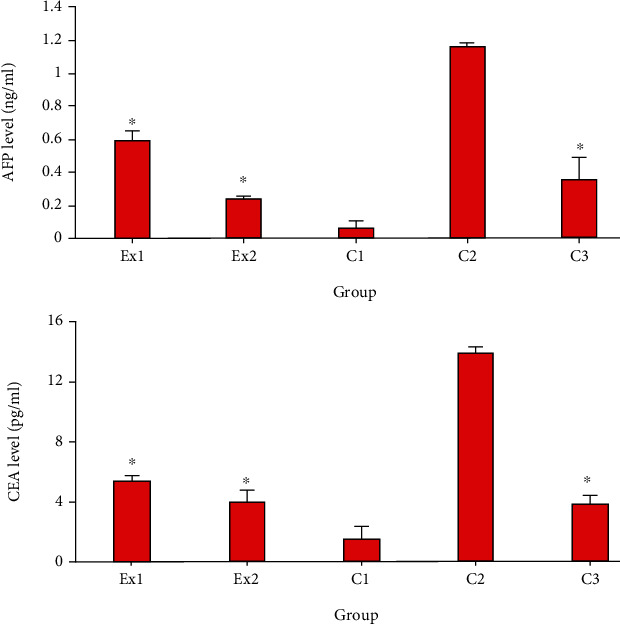
The serum level of alpha-fetoprotein(AFP) and carcinoembryonic antigen tumor (CEA) in EST-bearing mice treated with royal jelly (RJ) at the doses of 200 (Ex1) and 400 (Ex2) mg/kg when compared with (i) non-EST and nontreated mice (C1); (ii) EST mice receiving the normal saline (C2); (iii) EST mice treated with CP (50 mg/kg) (C3). Data are expressed as the mean ± SD (*n* = 8). ^∗^*p* < 0.001 significant difference compared with C2 group.

Today, it has been proven that the elevated serum level of AFP and CEA indicate liver and renal tissue damage during cancer [36]. The obtained results revealed that in the mice of control group of C2, the serum level of CEA and AFP was significantly elevated in comparison with the mice of C1 group. Nevertheless, the level of CEA and AFP was significantly (*p* < 0.001) decreased in the EST-suffering mice treated with RJ at the doses of 200 and 400 mg/kg when compared with the mice of C1 group (Figure 2).

**Figure 3:**
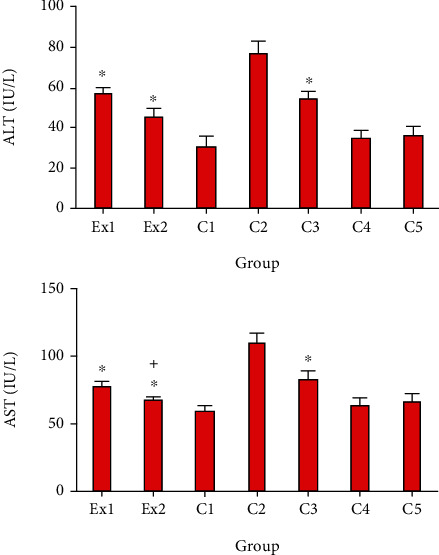
The serum level of alanine aminotransferase (ALT) and aspartate aminotransferase (AST) in EST-bearing mice treated with royal jelly (RJ). Non-EST and nontreated mice (C1); EST mice receiving the normal saline (C2); EST mice treated with CP (50 mg/kg) intraperitoneally once a day for 3 days (C3); non-EST mice treated with RJ 200 mg/kg orally once a day for 2 weeks (C4); non-EST mice treated with RJ 400 mg/kg orally once a day for 2 weeks (C5); EST mice treated with RJ 200 mg/kg orally once a day for 2 weeks (E1); EST mice treated with RJ 400 mg/kg orally once a day for 2 weeks (E2) (*n* = 8). ^∗^*p* < 0.001 significant difference compared with C2 group; + *p* < 0.001 significant difference compared with C3 group.

Due to the potential effect of cancer cells on the metabolism and function of liver cells and subsequently increased serum level of liver enzymes [37], we decided to measure the serum level of AST in the EST-bearing mice after treatment with RJ. Similar to the previous studies [38, 39], our findings revealed that the serum level of AST and ALT remarkably elevated in the mice of C2 group; representing that EST triggered severe hepatocellular damage. Whereas, in the EST-suffering mice treating with the RJ at the doses of 200 and 400 mg/kg the level of ALT and AST was meaningfully (*p* < 0.001) collapsed compared with the mice of C2 group (Figure 3). With respect to the toxicity profile of RJ, our results exhibited that there was no significant difference between the serum level liver and kidney enzymes in healthy mice and EST mice treated with RJ at the doses 200 and 400 mg/kg for two weeks.

**Figure 4:**
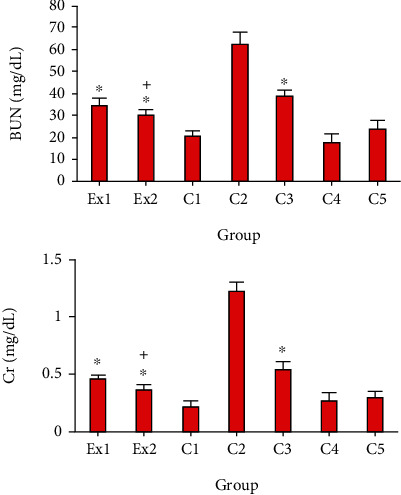
The serum level of blood urea nitrogen (BUN) and creatinine (Cr) in EST-suffering mice treated with royal jelly (RJ). Non-EST and nontreated mice (C1); EST mice receiving the normal saline (C2); EST mice treated with CP (50 mg/kg) intraperitoneally once a day for 3 days (C3); non-EST mice treated with RJ 200 mg/kg orally once a day for 2 weeks (C4); non-EST mice treated with RJ 400 mg/kg orally once a day for 2 weeks (C5); EST mice treated with RJ 200 mg/kg orally once a day for 2 weeks (Ex1); EST mice treated with RJ 400 mg/kg orally once a day for 2 weeks (Ex2) (*n* = 8). ^∗^*p* < 0.001 significant difference compared with C2 group; + *p* < 0.001 significant difference compared with C3 group.

As previously reported, the EST impairs the renal function and results in increasing the blood BUN and Cr [40, 41]. In consistent with previous investigations, Figure 4 indicated that the serum level of BUN and Cr considerably raised in in the EST-suffering mice in control group of C2; conversely, treatment of the EST-suffering mice with RJ at the doses of 200 and 400 mg/kg the level of BUN and Cr was meaningfully (*p* < 0.001) declined compared with the mice of C2 group.

Oxidative stress is one of the most important factors in the initiation and progression of cancer through increasing mutations and damage in DNA, genome variation, and inhibition of cell multiplying, etc. [42]. Studies shows that antioxidants agents especially those extracted from natural products are potentially able to interfere with carcinogenesis and preserve human beings from damages of oxidative stress [43–47].

Considering the effects of royal jelly on anticancer agentinduced toxicities, Amirshahi et al. have demonstrated the protective effect of royal jelly by improving the serum levels of testosterone and sperm parameters in bleomycin-induced male rats [48]. Karadeniz et al. reported that royal jelly modulates oxidative stress, apoptosis, and histological alterations in the liver and kidneys of rats treated with cisplatin [49]. In the study consucted by Malekinejad et al., the results showed the cardioprotective effect of royal jelly on paclitaxel-induced cardiotoxicity in rats by conferred protection against histopathological and biochemical alterations [50]. Another study conducted by Kaynar et al. revealed that royal jelly suppressed methotrexate-induced systemic oxidative stress and damage to small intestine in rats through an increase in the activities of antioxidant factors [51].

**Figure 5:**
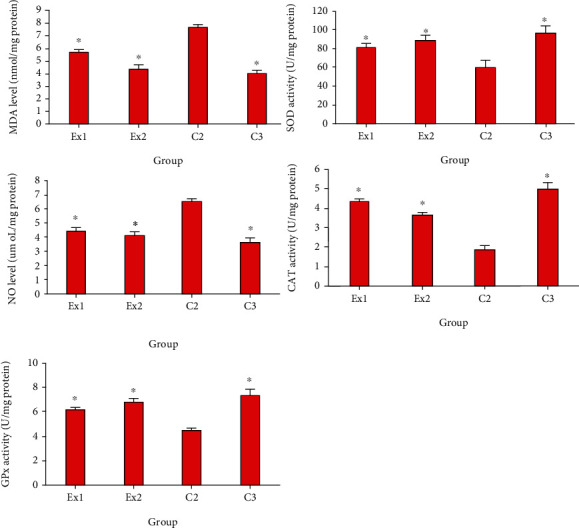
The tumor level of the malondialdehyde (MDA), nitric oxide (NO), glutathione reductase (GR), glutathione peroxidase (GPx), catalase enzyme (CAT), and superoxide dismutase enzyme activity (SOD) in EST-suffering mice treated with royal jelly (RJ). EST mice receiving the normal saline (C2); EST mice treated with CP (50 mg/kg) intraperitoneally once a day for 3 days (C3); EST mice treated with RJ 200 mg/kg orally once a day for 2 weeks (Ex1); EST mice treated with RJ 400 mg/kg orally once a day for 2 weeks (Ex2). ^∗^*p* < 0.001 significant difference compared with C2 group. Data are expressed as the mean ± SD (*n* = 8).

As shown in Figure 5, the obtained findings exhibited that the tumor level of MDA and NO was meaningfully raised; but the level of GPx, CAT, and SOD was considerably reduced in the mice of C2 group. Conversely, RJ at the doses of 200 and 400 mg/kg/day considerably (*p* < 0.01) declined the expansion in the LPO and NO as well as raised (*p* < 0.05) the level of GPx, CAT, and SOD. Similarly, Zhang et al. demonstrated that RJ at the doses of 0.5 and 1.5 g/kg meaningfully increased the SOD and total antioxidant capacity (T-AOC) in the 4 T1-suffering mice [31]. Moreover, previous studies have revealed the antioxidant effects of RJ by DPPH-based radical scavenging with IC_50_ value ranging 150 to 219 *μ*g/g for various royal jelly samples [32]. In line with our results, Abdel-Hafez et al. have demonstrated the protective effect of royal jelly against cyclophosphamide induced prostatic damage in male albino rats through the reducing of NO and MDA as well as increasing of GPx, GST, and SOD [52].

**Figure 6:**
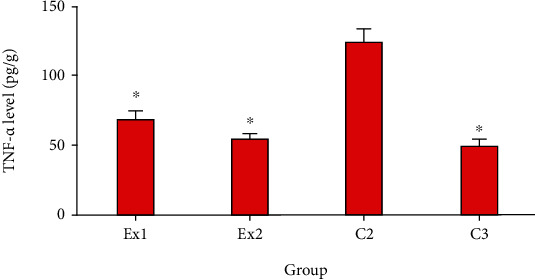
The tumor level of the TNF-*α* in EST-bearing mice treated with royal jelly (RJ) EST mice receiving the normal saline (C2); EST mice treated with CP (50 mg/kg) intraperitoneally once a day for 3 days (C3); EST mice treated with RJ 200 mg/kg orally once a day for 2 weeks (Ex1); EST mice treated with RJ 400 mg/kg orally once a day for 2 weeks (Ex2). ^∗^*p* < 0.001 significant difference compared with C2 group. Data are expressed as the mean ± SD (*n* = 8).

Based on previous studies, cancer cells can damage normal tissues and result in hypoxia via two main methods including (i) mechanical injury of the tumor and (ii) stimulating the secretion of some proinflammatory cytokines [53]. As shown in Figure 6, the obtained results confirmed that the level of TNF-*α* in the EST-suffering mice in C2 group was considerably (*p* < 0.001) raised; nevertheless, treatment of the EST-suffering mice with RJ at the doses of 200 and 400 mg/kg/day meaningfully (*p* < 0.05) declined the level of TNF-*α* in mice. In line with our results, Miyata et al. have exhibited that oral administration of capsules having 900 mg royal jelly significantly decreased the serum levels of TNF-*α* in patients receiving capsules having 900 mg royal jelly [30].

**Figure 7:**
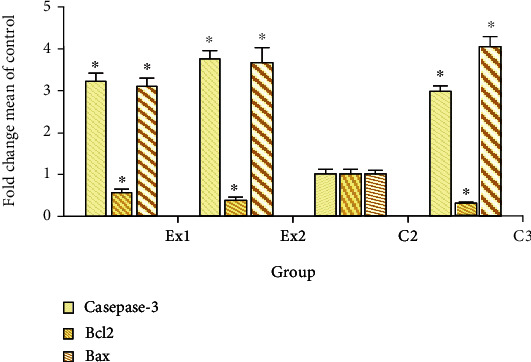
The expression level of the caspase-3, Bcl-2, and Bax genes in EST-bearing mice treated with royal jelly (RJ). EST mice receiving the normal saline (C2); EST mice treated with CP (50 mg/kg) intraperitoneally once a day for 3 days (C3); EST mice treated with RJ 200 mg/kg orally once a day for 2 weeks (Ex1); EST mice treated with RJ 400 mg/kg orally once a day for 2 weeks (Ex2). ^∗^*p* < 0.001 significant difference compared with C2 group. Data are expressed as the mean ± SD (*n* = 8).

Apoptosis or programmed cell death is well known as one of the key factors in various phases of a living organism's biological evolution and which in case of irregular and abnormal activity, it results in various serious diseases [54]. Apoptosis inhibition is one of the main routes in tumorigenesis and cancers which is essential for cancer cells to continue their uncontrollable proliferating. Hence, induction and elevation of apoptosis is a standard target to discover new anticancer agents [55].  Figure 7 exhibited that the expression level of caspase-3 gene was significantly (*p* < 0.001) upregulated in tumor tissues, with 3.22 and 3.76 fold after treatment of the EST-suffering mice with RJ at the at the doses of 200 and 400 mg/kg, respectively. Likewise, the expression level of Bax gene was meaningfully (*p* < 0.001) upregulated in tumor tissues, by 3.11 and 3.98 fold after treatment of the EST-bearing mice with RJ at the doses of 200 and 400 mg/kg, respectively. The findings of quantitative real-time PCR displayed that the expression level of Bcl2 was considerably (*p* < 0.05) downregulated in the tumor after treatment of the EST-suffering mice with RJ at the doses of 200 and 400 mg/kg, respectively. Similarly, Aslan et al. and Ali et al., have demonstrated that royal jelly elevates the caspase and Bcl-2 expression level and subsequently declined the muscle tissue injury induced with fluoride in rats [56, 57].

## 4. Conclusion

The findings of the current study revealed that oral administration of royal jelly especially at the doses of 200 and 400 mg/kg exhibited promising antitumor effects against EST in mice and might be proposed as a novel anticancer agent against tumor; however, additional surveys especially in clinical setting are necessary to approve these findings.

## Figures and Tables

**Table 1:** Sequence of primers of used for real-time PCR.

Amplicon	Primers	Sequence (5′–3′)
Bax	FR	GGCTGGACACTGGACTTCCTGGTGAGGACTCCAGCCACAA
Bcl-2	FR	CATGCCAAGAGGGAAACACCAGAA GTGCTTTGCATTCTTGGA TGAGGG
Caspase-3	FR	TTCATTATTCAGGCCTGCCGAGGTTCTGACAGGCCATGTCATCCTCA
*β*-Actin	FR	GTGACGTTGACATCCGTAAAGAGCCGGACTCATCGTACTCC

**Table 2:** The results of measurement of the secondary metabolites contents of RJ.

Total content	Test	Amount
Phenolic	Folin–Ciocalteau's reagent colorimetric	96.3 ± 0.31 mg GEA/g DW
Flavonoids	Aluminum chloride (AlCl_3_ 2%) colorimetric	2.85 ± 0.026 mg QE/g DW
Protein	Bradford method	11.3%

**Table 3:** Effect of various doses of royal jelly (RJ) of tumor volume and tumor inhibition rate.

Group	Tumor volume (g)	% of inhibition
RJ 200 mg/kg	1.54 ± 0.04	50.6
RJ 400 mg/kg	0.86 ± 0.22	72.4
C2	3.12 ± 0.12	—
C3	0.96 ± 0.03	69.2

C2: EST mice receiving the normal saline; C3: EST mice treated with CP (50 mg/kg) intraperitoneally once a day for 3 days (C3). Data are expressed as the *mean* ± *SD* (*n* = 3).

## Data Availability

All data generated or analyzed during this study are included in this published article.

## Abbreviations

EST: Ehrlich solid tumors
AFP: Alpha-fetoprotein
CAE: Carcinoembryonic antigen tumor
LPO: Lipid peroxidation
NO: Nitric oxide
Gpx: Glutathione peroxidase
CAT: Catalase enzyme
SOD: Superoxide dismutase enzyme activity
TNF-*α*: Tumor necrosis factor alpha level
ALT: Alanine aminotransferase
AST: Aspartate aminotransferase
Cr: Creatinine
BUN: Blood urea nitrogen.
